# Researching urban diversity and the (re-)production of whiteness: reflections on the purchase and challenges of sensory methods

**DOI:** 10.3389/fsoc.2025.1512271

**Published:** 2025-05-07

**Authors:** Christine Barwick-Gross, Jari Chollet, Christy Kulz

**Affiliations:** ^1^Department of Urban and Regional Sociology, Humboldt University of Berlin, Berlin, Germany; ^2^Department of Planning and Architectural Sociology, Technical University of Berlin, Berlin, Germany

**Keywords:** race, whiteness, sensory research, smell, sound, cities, sensory urbanism, migration

## Abstract

In this paper, we reflect on the purchase of sensory methodologies to research urban diversity and the (re-)production of whiteness. In the social sciences, scholars commonly rely on visual methods, using the ‘body as text’ (Stoller, 1997). Based on more recent advances in urban and migration studies, we seek to move beyond this Eurocentric focus, by asking how urban diversity is experienced through sounds and through smells. How individuals experience sensory inputs such as sounds and smells, and how they make sense of them, feeds into processes of boundary making and boundary crossing. The urban space is a prime context to study such processes, given that cities’ dense character and high diversity provide their residents with endless sensory stimuli. Based on sensory research in a highly diverse street in Berlin, we reflect on how smells and sounds contribute to creating hierarchies between (groups of) people, how they contribute to feelings of local belonging and home, or feelings of being out of place. We also reflect on the challenges of applying sensory methods. These refer to the relationship between participants’ embodied experiences and how they made sense of those discursively in group discussions; and about the implications of doing research on a highly mediatized street. With our focus on micro interactions, the (re-) production of space, and diversity, our findings add to the emerging field of ‘sensory urbanism’.

## Introduction

In the social sciences, just as in other neighboring disciplines such as anthropology or human geography, methodologies predominantly focus on the visual and produce texts. Regarding the topic of this paper, namely the experience of urban diversity and related processes of belonging and exclusion, scholars commonly use various interviews, participant observation, or discourse analysis. However, conceiving of the “body primarily as a text that can be read and analyzed” (Stoller, 1997: xiv) is a Eurocentric practice that disregards senses other than the visual (cf. Osbourne, 2021). To counter this prevailing approach, Stoller (2004: 820) argues for “sensuous epistemologies,” stressing the importance to attend in more detail to the “relationship between sensuous perception, power, and knowledge”. This not only gives new and relevant insights into the analysis of social problems, but also encourages the researcher “to rethink their scholarly being-in-the-world” (Stoller, 2004: 820).

The words of Stoller and other scholars calling for more attention to the sensory are slowly finding an echo in Western scholarship, particularly after the so-called “sensory turn” (Eisewicht et al., 2021). Attending to the senses places attention on people’s embodied experiences. As Wise (2010: 933) argues, the senses “are the means by which we apprehend our environment and these senses are habituated, linking our bodies to experiences, sensations and emotions.” Scholarship that attends to people’s sensorial experiences is tightly connected to the study of memory, affect and emotions (LaBelle, 2010). As an embodied experience, situated temporally and spatially, sensory experiences are deeply influenced by an individual’s physical capacities, their biography and cultural embeddedness (Le Breton, 2017).

Several scholars have highlighted the role of the senses - other than sight - in the making of race (Smith, 2006; Sekimoto and Brown, 2020). While race is often understood as a purely visual construct, the multisensorial dimension of race is often overlooked (Smith, 2006, Sekimoto and Brown, 2020). As a matter of fact the construction of race through other senses can already be seen in the colonial period, where an increasingly racially mixed population made it necessary to rely on other indicators than sight for upholding racial difference (Smith, 2006). Marking race through smell, sound, taste or touch always played a part in biologizing and naturalizing the social construct of race, and therefore needs to be examined for a thorough understanding of race and racialization (Smith, 2006).

Expanding on the notion of race as a “sliding signifier” (Hall, 2017: 79) that is deeply influenced by its context, the understanding of such a context has to be enriched with a sensual dimension. Race can then be understood as a “bodily, affective, and sensorial event - something that happens, rather than something that is” (Sekimoto and Brown, 2020: 3). This understanding helps to unpack the processes and mechanisms through which race is made real in people’s everyday lives and experiences. Race is constructed and made real through the senses, and at the same time one’s positioning in the racial hierarchy impacts how sensory inputs are experienced and interpreted. As three white, highly educated researchers from the Global North, it is particularly important that we reflexively recognize our position in the field and how this relates more widely to the hegemony of whiteness [see Bhatt (2016)].

One field in which the senses have found more attention is in ‘sensory urbanism’. Very broadly, sensory urbanism focuses on “the extent to which the socio-spatial order of cities is a sensory order” (Jaffe et al., 2019: 1017). The emerging scholarship in this field focuses on the experience and interpretation of city dwellers’ “embodied sensations” (Jaffe et al., 2019), and how these sensations are linked to cities’ materialities as well as to processes of urban planning, regulations, and governance. Several studies in this field address the “visceral micro-politics of urban exclusion” (Pow, 2017) that occurs through “sensory othering” (Pow, 2017), particularly in processes of urban/neighborhood transformation such as gentrification or increasing migration-induced diversity (Fiore, 2023a; Wise, 2010; Pow, 2017). Overall, these studies point to how sensory experiences are based on one’s racial positioning, how sensory moral orders are used to exclude certain groups of city dwellers, and, relatedly, how the sensory is used to (re-)produce hierarchies between groups, based particularly on race, ethnicity, and socioeconomic background. Sensory urbanism thus provides new insights into the study of urban inequalities.

Our study intervenes in this literature, asking how urban diversity is experienced through sound and smell, and how whiteness is (re-)produced in the process. Migration-induced diversity is one of the major transformations that cities and neighborhoods are experiencing (Vertovec, 2007); this transformation often entails conflicts and negotiations around the commercial use of space, around claims to belonging and the making of home. While many studies use the ‘superdiversity’ concept to examine ways of living together (Wessendorf, 2014; Vertovec, 2007), these studies have been criticized for their lack of attention to issues of power, particularly as related to race/racism (Barwick-Gross, 2024; Beebeejaun, 2022). While our study is set in a context that is highly diverse, our interest is not only how this diversity is experienced through sound and smell, but also how sensory experiences and their interpretations reproduce racial hierarchies and hence urban inequality.

Our main interest in this paper is epistemological and methodological: While we give insights into the results of a study we conducted together in Berlin, these results are primarily used to invite reflection on the sensory methods that we explored, highlighting what they can do, e.g., in terms of producing new knowledge, but also the perceived limits or need to further develop our intervention in the field. As Moretti (2021: 308) argues, “a sensuous and embodied orientation to scholarship encourages different epistemological approaches, opening up avenues for learning, remembering, and thinking otherwise”. In the following, we will present two fields that give insights to our research interest: first, we focus on the senses and the (re-)production of race and whiteness. In line with the focus of our study, we thereby concentrate on smell and sound. The second field we draw from is sensory urbanism and the role of the senses for the reproduction of urban inequality. We then provide a description of how we approached the topic methodologically through a study of a diverse street in Berlin, Germany, followed by insights from our research which highlights the purchase, but also the limits, of the sensory methodologies we applied.

### Creating and (re-)producing racial hierarchies through the senses

Acknowledging that sensual perception is dependent on its cultural context enables a perspective on the role of the senses in engaging with each other. Through regulating how and what can or should be sensed, societal norms are reproduced, which leads Howes and Classen (2014: 5) to claim a “politics of the senses”. Senses create social boundaries as well as hierarchies “through acts of marking, excluding, punishing or exalting particular individuals and groups” (Howes and Classen, 2014: 66). Largey and Watson (1972: 1025), who proposed a ‘Sociology of Odor’ as early as 1972, pointed to how the “alleged malodor” of supposed others serves as “a crucial component” in categorizing others. Le Breton calls smell the “sense par excellence of racist or class prejudice,” highlighting that “People’s olfactory designation offers a moral status upon them” (Le Breton, 2017: 164.). To illustrate, Kettler (2020) describes how African subjects were categorized as foul smelling, an “olfactory racism” that played into the legitimization of hierarchies between (white) Europeans and Black Africans who were then called ‘savages’ and traded as slaves. Judging the smell of others as bad thus reflects and reinstates hierarchies that already exist between societal groups. Put differently, social hierarchies are translated into olfactory hierarchies. Thereby, subjects are sensorily ‘othered’ by marking them as foul-smelling. This “sensual marking” is embedded in “hegemonic, seemingly odourless smellscapes” (Liebelt, 2023: no page), meaning that whiteness is connected to a norm of odourlessness.

The same is true for sound, although its cultural production is generally more accepted than it is the case for smell, touch or taste, which are often considered private (Howes and Classen, 2014). Differentiating between what is pleasant sound versus noise/nuisance, the latter is usually attributed to ‘the other’, such as racialized or migrantized individuals, but also the lower classes. As Stoever (2016: 4) shows in her historical account of the ‘Sonic color line’, based on archival research in the US, “listening operates as an organ of racial discernment, categorization, and resistance in the shadow of vision’s alleged cultural dominance”. She describes the relevance of sound for how racial identities are imposed on others, but also how such impositions can be resisted through sound. For Stoever, “The sonic color line describes the process of racializing sound— how and why certain bodies are expected to produce, desire, and live amongst particular sounds— and its product, the hierarchical division sounded between “whiteness” and ‘blackness’” (7). She uses the figure of the listening ear to describe the dominant listening practices which form biographically and culturally, and enforce the adherence to dominant sonic norms. This echoes Kanngieser (2023: 14) argument that listening is political, and that “class, economics, culture, race, and gender affect our communication”.

The equivalent to attributing foul odor to marginalized minorities lies in how certain sounds are categorized as noise or nuisance. Noise, as Atkinson (2007: 6) argues, is “sound which is out of place.” Again, what is deemed to be in or out of place is highly subjective and culturally coded. In this respect, Stoever (2016: 12) argues that whiteness is commonly thought of as inaudible, as “correct speech”, while the sounds of Black people (and other minorities) are categorized as exotic, unruly noise. Hence her argument that “white Americans often feel entitled to respect for their sensibilities, sensitivities, and tastes, and to their implicit, sometimes violent, control over the soundscape of an ostensibly free, open, and public space” (Stoever, 2016: 2). Generally, one’s ideas about race shape what and how one hears, and vice versa.

It is important to stress that different sensory stimuli can not be neatly differentiated, but have to be understood as part of an interactive sensory experience. In Western societies, given the predominant role of audiovisual media, a strong connection between sight and sound is often acknowledged, while the connection between other senses might easily be overlooked. The term of “intersensoriality” (Howes and Classen, 2014: 5) therefore emphasizes the “manifold relations among the different senses” (Howes and Classen, 2014). Especially due to the learned fixation on the visual as a means of understanding our environment, what one sees can strongly affect what is heard or smelled.

### Sensory urbanism and the reproduction of race

The outlined relations between the sensory and the production of race and whiteness are highly dependent on spatial and temporal contexts. Regarding the former, cities provide a particularly relevant context, as they provide – or confront – their inhabitants with endless sensory stimuli which are hard to escape, something that Georg Simmel already pointed out in the early 1900s (cf. Frisby and Featherstone, 1997). Furthermore, the city as a highly diverse space through migration-induced diversity, but also gender, sexuality, lifestyles, etc., is a prime place to study everyday practices of city dwellers, and how they use ‘sensory othering’ to draw boundaries between different groups of urban inhabitants and actors, i.e., the micro-politics of exclusion (Pow, 2017).

As scholars of sensory urbanism have highlighted, attending to the senses in the city gives insights into “the governance of urban sensation” (Jaffe et al., 2019), as well as into “how everyday practices and discourses of sensory ‘othering’ reproduce urban inequalities” (Jaffe et al., 2019: 1017). The first dimension (governance) emphasizes the side of planning, regulations, and policy and how the material environment, which is a result of urban planning and policymaking, impacts on the sensory regimes and sensory orders of cities. The senses, as Low (2015: 296) argues, “mediate one’s engagement with urban growth and development, hence rendering insights into the multi-sensory character of urbanity.”

While the material environment of the city marks places and can create division, sensory inputs such as sound and smell can transgress these spatial markers. As Aceska and Doughty (2024) have shown by reflecting on the case of the divided city of Mostar (Bosnia-Herzegovina), conceptions of us and them can be transgressed through the senses. Using sounds related to religion, i.e., the call for prayer and church bells, the authors show how such transgressions can lead to urban conflict, but also to new ways of being together. In many European cities, “The call to prayer has featured strongly in the recent public debates (…) about what kind of diversity can claim a presence in public space” (Aceska and Doughty, 2024: 5). In this way, regulating sound can be a way of regulating diversity, of who can claim (aural) presence in the city and who cannot.

Based on a sensory ethnography of a gentrifying neighborhood in Rome, Fiore shows (Fiore, 2023a: 276) “how the sensuous-material textures of place enabled or obstructed residents’ emplacement”. The perception of urban dirt in a local park was tightly connected by the in-moving white gentrifiers with the presence of (partly homeless) immigrants in the park who were blamed for leaving their waste and hence violating the sensory order that these gentrifiers would expect to being able to feel at home, to feel emplaced. Instead of directing the blame toward the city and its abandonment of the neighborhood, the “conflation of immigrants and waste in a negative stereotype points to how racialized perceptions of urban dirt and disorder can become tools for exclusionary placemaking, especially in ethnically diverse neighborhoods” (Fiore, 2023a: 274).

These examples show that sensory experiences do not just happen in the city, but are influenced by the built environment, political regulations, urban planning and policies. Foregrounding the role of sound and smell is one way - so far largely neglected - to highlight how bodies are situated “within the context of policies and other means of governance and control” (Aceska et al., 2024: 12), and how such policies and control are experienced ontologically.

Regarding the “visceral micro-politics” of everyday practices, the sensory is crucial in the (re-)production of symbolic and social boundaries, but also in challenging and overcoming such boundaries. Such boundary-making is tightly connected to claims to belonging, i.e., a sense of being in place, which is influenced by and mediated through the senses. To illustrate, Lisiak et al. (2021) examines the effects of the increased presence of immigrants in certain London neighborhoods. They show how “The sense of discomfort or disorientation produced by a proximity to cultural difference is about a loss of control and a seizure of anxiety in the heart of whiteness” (Lisiak et al., 2021: 264), which in their case is mirrored by adverse reactions toward the sounds of foreign languages, or, more generally, sounds that are categorized as noise/nuisance and as creating discomfort.

Also focusing on sounds as nuisance, Fiore (2023b) highlights a common conflict in gentrifying neighborhoods, namely that in-moving (white) gentrifiers feel disturbed by the sonic practices of long-term residents, residents who are marginalized due to their migrant or racial background (cf. also Summers, 2021). Set in an Amsterdam neighborhood, the new residents complain about Moroccan-Dutch youth, a group that is generally portrayed in media and political discourse as a nuisance and a security threat. This “cultivated sensory practice” (Fiore, 2023b: 25), based on a racialized nuisance/security discourse, leads to a “sensory criminalization” (ibid.: 24), which designates certain sonic practices in public spaces as morally acceptable versus unacceptable. Ultimately, the “sensitivity to a heavily racialized kind of nuisance is (…) elevated to an organizing lens for white place-making” (ibid.: 25).

Focusing on another type of urban transformation, namely a stronger presence of immigrants in a neighborhood, Wise highlights the strong affective and emotional responses from long-term white residents. Based on the example of an Australian suburb which was populated primarily by white Australians, but has recently seen a rise of migrants from China, Wise (2010: 932) highlights how “The bodily, muscular, visceral quality of place relations can, under conditions of radical change, produce affective counter-responses that are all the more emotionally intense, filled with bodily revulsion and neurotic bitterness”. These reactions were based partly on the supposed bad smell that the newcomers brought with them, often related to food. The affective reactions were so strong that the author talks about “neurotic emotions” that were “associated with nostalgia, change, and the presence of otherness” (Wise, 2010: 932). Thus, changes in one’s home can clearly lead to conflicts; they can lead to painful emotions, both of newcomers and of the (often white) long term residents; these latter often feel that their home is taken away from them, a sentiment that is commonly expressed in racist and exclusionary ways, and sometimes as shame.

While many studies focus on boundary drawing between groups of city inhabitants, a focus on the senses also allows us to register the crossings of such boundaries. Particularly in highly diverse urban spaces, it is necessary to examine the “banal aspects of everyday life that are overlooked by conventional modes of sociology” (Rhys-Taylor, 2013: 394). Rhys-Taylor’s olfactory and gustatory ethnography of a London street market shows the role of the senses for processes of transculturalization, which characterizes “the everyday life of inner city London” (Rhys-Taylor, 2013: 394) – and so many other, diverse, cities.

Given this theoretical background, we will explore the following questions, based on a project about the sensory experiences of urban diversity in Berlin: How is urban diversity experienced through the senses? What is the role of the senses in drawing boundaries between people, and how does this reproduce race and whiteness?

### Methodological intervention

Based on the aforementioned literature, we set up a project to test the purchase – and the limits – of sensory methodologies to study urban diversity, including the drawings of boundaries based on race and whiteness, but also migration/ethnicity, age, gender, and class. As the project was rather open and explicitly designed to test a variety of methods to capture sensory experiences, our methods developed across the duration of the project, mostly in reaction to the limits of certain methods that we noted.

**Figure 1:**
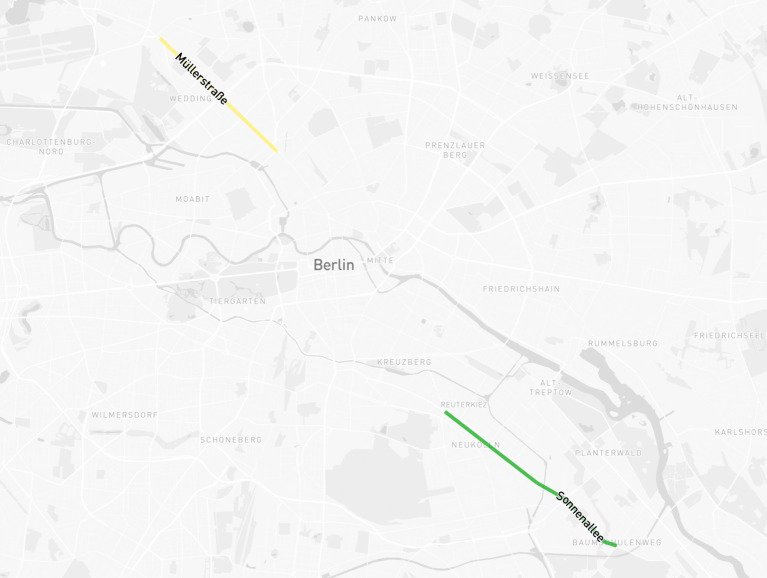
Locations of Müllerstraße (Wedding) and Sonnenallee (Neukölln). Map created by Jari Chollet, with Mapbox.

Our research took place between April 2023 and August 2024 and was set in two streets in Berlin: Müllerstraße in Wedding and Sonnenallee in Neukölln (Figure 1).

As this paper concentrates on findings from Sonnenallee, we will only describe this context. Sonnenallee is regularly in the center of local and national media discussions around migration and integration. Most recently Sonnenallee was in the media spotlight for pro-Palestinian protests following the massacre on 7th October 2023 (Sharma, 2024). Sonnenallee functions as main avenue and shopping street in Neukölln (next to two parallel streets), which heightens its sensory intensity. This is especially true for the northern part of Sonnenallee (between Hermannplatz and the metro station “Sonnenallee”), which our research focused on.

Sonneallee’s image is characterized by immigration. During the guest worker period, many Turkish immigrants moved to Northern Neukölln. As a neighborhood next to the Berlin Wall, rents were rather cheap. In the 1970s, refugees and asylum seekers from Lebanon, but also stateless Palestinians, often with highly precarious residence titles, increasingly settled in Neukölln. From 2015, Sonnenallee has functioned as an ‘arrival space’ for Syrian refugees. The neighborhoods around Sonneallee are also characterized by intra-EU migration from Eastern Europe and Southern Europe. A high percentage of the population on and around Sonnenallee thus has its own or parental migration history.

The neighborhood is also socially diverse, characterized by clear signs of gentrification, including soaring rents, but also continuing high levels of segregation, particularly in schools. The changing population structure as well as gentrification have also led to a change in the business infrastructure of Sonnenallee, which has changed from a predominantly Turkish street to an ‘Arab street’ (which is how it is colloquially referred to in Berlin). While many Turkish businesses had to close during the 1990s, due to economic downturn, the increased immigration from Syrian refugees has led to a revitalization of the street, and a re-opening of shops that have been vacant for a long time^1^. Overall, thus, Sonneallee is a highly diverse street, across several dimensions, and therefore a fitting context to examine the sensory experiences of diversity.

Sensory methods are often mobile methods, based on experiencing a place through walking (Bates and Rhys-Taylor, 2017; Degen and Rose, 2012). Our research thus started with auto-ethnographic sound walks and smell walks, to get a sense of how we perceived sensory stimuli in both streets. Given the dominance of the visual, we also had to learn to listen, to smell, and to express sensory input in words – often a challenging exercise given the restricted vocabulary that we (in the West) have to name sounds and smells (Le Breton, 2017).

**Figure 2:**
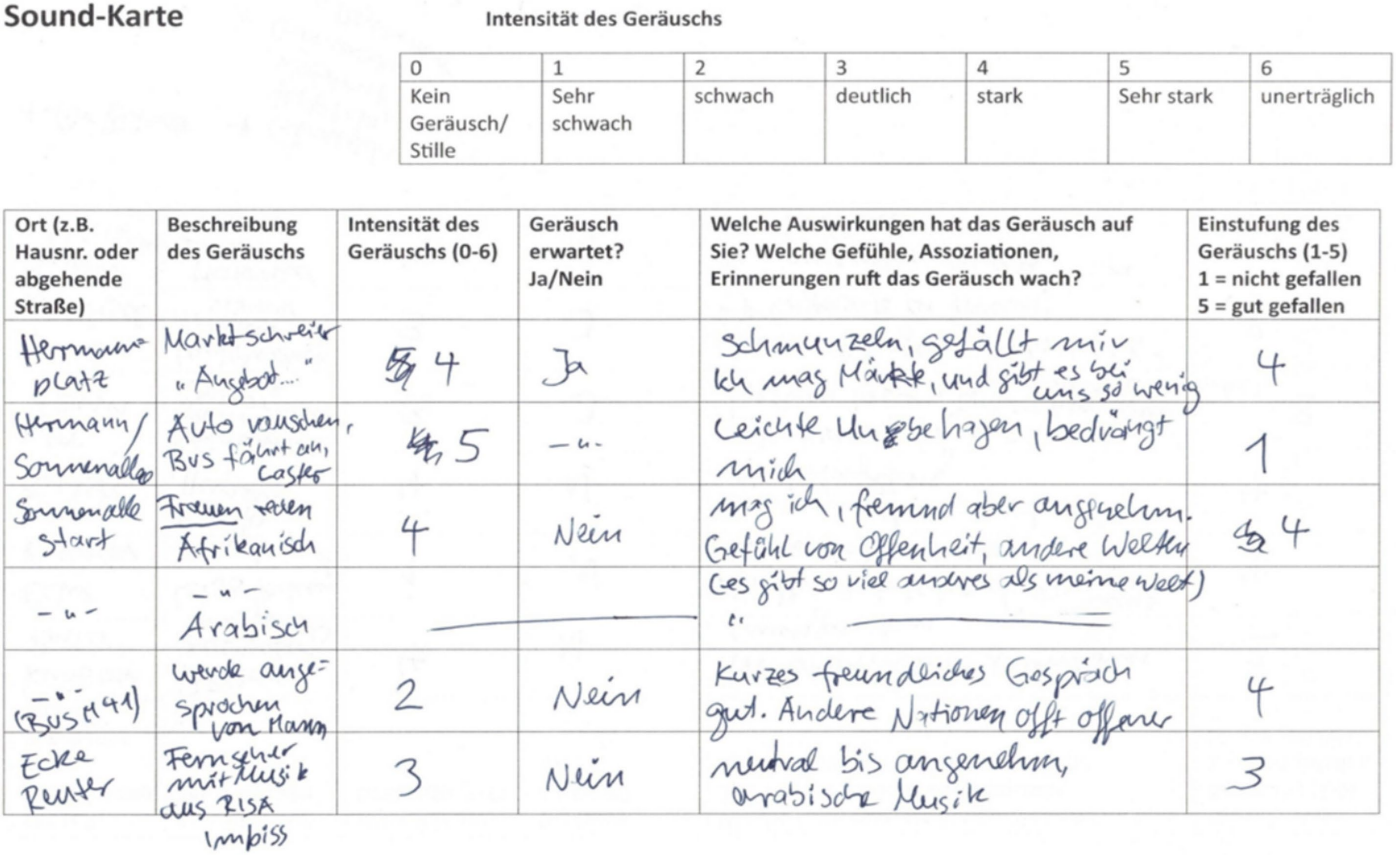
Example of a sound map (in German).

In a second step, we looked for participants for smellwalks and soundwalks. These walks were conducted in groups. To start, every participant was presented with a question about their associations with the street, which they noted on a piece of paper. In a second step, they individually conducted either a smellwalk or a soundwalk, equipped with a sound map or smell map (Figure 2), respectively, to note the place of the sound/smell, its intensity (scale from 1–6), the naming of the sound/smell, whether it was expected or not, the feelings, emotions, memories it evokes, and whether the sound/smell was pleasant or unpleasant (scale from 1–5). After the walks which lasted roughly one hour, we met in a café for a group discussion about the participants’ experience of the walk. These interviews lasted for up to 1,5 h and served to get general feedback, but also to compare participants’ experiences and interpretations, and to provide a forum for exchange.

The participants were recruited through different channels: neighborhood platforms, student lists at our universities, and by contacting friends and neighbors. The participants differed regarding their place of residence, i.e., whether they lived in the area or not, age, and gender. However, most participants were from a rather privileged background, and they were mostly read as white (although some of the student participants were born abroad and had migrated to Germany, or one of their parents migrated). To get a more diverse sample of participants, particularly regarding our interest in the production of race and whiteness, we contacted local stakeholders, particularly employees of local community organizations. In total, we interviewed 25 participants in Neukölln of which 23 also did a smell or a sound walk. Among the participants, 15 had own or parental migration history.

Lastly, we also conducted semi-structured interviews with local shopkeepers. In these interviews, we focused primarily on how the shopkeepers perceive the street and its diversity. While we also asked how they would characterize the streets regarding sounds and smells, these questions did not work well. This was partly due to language issues, particularly in Sonnenallee, where several shopkeepers did not speak German fluently, and we were not able to provide a translator. We return to this point in our reflections.

In the following, we will provide some insights into the results. Again, our aim is not to provide an extensive analysis of all smell and sound walks, but to use the examples to reflect on the advantages and the limits of the methodologies that we applied throughout the course of our research.

### Insights from the sensory walks

Following our discussion of the benefits of engaging the senses when researching racialization processes in urban spaces, this section will discuss how the methodological approaches used could (or could not) contribute to gaining new insights into the production (or crossing) of boundaries in urban space. For the sake of our argument, we do not present a detailed analysis, but rather highlight examples that provoke thoughts about how we could further develop these methods. Extracts from the interviews and collected data were translated into English, since most walks originally took place in German. All names have been changed.

#### Smelling ‘the other’

Charlie, a young, white-read master’s student, joined a smellwalk on Sonnenallee. Living in Berlin’s North-east, she does not use the street in everyday life. She has a certain image of Sonnenallee, associated with topics such as “cultural diversity,” “(chaotic) traffic” and “food/culinary experience.” Her participation can be likened to that of a tourist, immersed in a rather unfamiliar landscape that evokes a “temporary experience of sensory unfamiliarity and disruption” (Jaffe et al., 2019: 1021). She does not have many previous memories of walking around in the street (cf. Degen and Rose, 2012), and her knowledge comes mostly from external sources. Charlie finds that the walk confirmed her prior view of Sonnenallee - a view (cf. Fiore, 2023a) based on the general image of Sonnenallee that is promoted in public, political and media discourse. Like other respondents, she links food smells to ideas of traveling, which orientalized and exoticized her sensory inputs: “… so I also had associations such as diverse food and multiculturalism [*Multikulti*] and then with the associations of traveling, the Orient and so on by the smell of things.” In other interviews, associations with “Orient,” “traveling” or “vacation” played a major role as well.

Charlie describes the overall smell on Sonnenallee as “stuffy,” which she explains as “the smell that is present in a warm, heated city with lots of concrete.” While this association speaks to a view of Sonnenallee smelling like any ‘heated city’, the actual smell examples she gathered paint a different picture. Here the smell of Sonnenallee does not appear as uniform and diffuse stuffy air, but is separated into distinct experiences of either “disgust” or “appetite.” While the contrast between disgust and appetite/hunger appears in other accounts, this dualism is most obvious in Charlie’s smell map. The smells she wrote down almost unanimously result in associations that either contain the words “disgust” (perfume, smoke and stuffy air) or “appetite/Orient/traveling” (spices and food). Charlie reflects on the quick change between intriguing and repulsive smells in the accompanying interview: “It was like that_ at one point: oh tasty, smell_ kind of, yeah spices or some food. And then some steps further, it was a kind of a stench in a way.”

As outlined before, smell perceptions reflect racial hierarchies through reproducing whiteness as an absence of smell, while at the same time marking ‘the other’ as smelly in the sense of having a strong odor. Charlie’s example shows how the street is perceived as having a foul smell, expressed through repeated associations of disgust. At the same time, her associations with positive smells also re-produce an otherness. Using associations such as “Orient” or “traveling” creates and reproduces an ‘otherness’, by locating the smells elsewhere. While they are positive, they do not necessarily belong to the locality. Placing people elsewhere is a typical way of migratizing them (Tudor, 2017), and this process can apply to sensory inputs. Connecting certain smells to a geographical elsewhere portrays parts of Sonnenallee as departing from the norms of mainstream society, which is tacitly positioned as white and located in Germany. As such, the smell of certain foods and spices is welcome, but it is interpreted as different, as being not from or of Berlin. The smells are idealized and exoticized - a typical process of othering, as described by Said (2019). hooks (1992: 21) writes about ‘eating the other’, as ethnicity seasons the ‘dull dish of mainstream white culture’, yet here exoticisation operates through ‘smelling’ the spicy other. Drawing boundaries and reproducing social hierarchies through smell, therefore, is not only linked to a projection of bad odor, but at the same time to an exoticizing discourse evolving from the perception of certain smells. Of course this does not necessarily entail that a certain odor was actually present, but the shown discursive factor in perception points to the complex interaction of media induced images and multiple senses. Given how sensing is cultivated (cf. Fiore and Caradonna, 2025), the alleged smells might actually result from *seeing* racialized bodies and connecting them to a certain discourse about the ‘smelly other’.

Charlie’s example not only highlights the process of othering through exoticizing smells, but shows the extreme categorization of smells as either repulsive or intriguing, with nothing in between. This dualistic response to smell as intriguing or repulsive ties to Stuart Hall’s description of racialization. Following Sander Gilman, Hall describes how a discourse of Othering uses a Manichean worldview to construct a binary between ‘us’ and ‘them’. Here all contradictions are projected onto the proclaimed ‘other’, splitting it into a ‘bad other’ and a ‘good other’ (Hall, 2000: 167ff). Thus, smell works to reproduce racial hierarchies by marking out the ‘other’ as either smelling badly or smelling ‘exotic’, in both cases reinforcing the idea of the ‘other’ as being ‘smelly’, i.e., having a smell in opposition to a white and supposedly odorless norm (Liebelt, 2023). We can see how these othering practices operate not only textually, but through sensory phenomena like smell and its association with another time or place. Çağlar (2016) draws on Fabian’s seminal text *Time and the Other* (1983) to describe how racialized minorities are continually placed in another place and time, even when they were born in Europe. She cites how this disallowance of coevalness or contemporaneity acts as an othering device, excluding and perpetually migrantizing ethnic others while reifying whiteness. Fiore and Caradonna (2025) make a similar point, talking about food tours offered by the Tropenmuseum in Amsterdam. Set in a traditional immigrant neighborhood that is experiencing processes of gentrification, these tours (which exist in similar fashion in Berlin) not only primarily provide happy stories of diversity, but they also created a “spatiotemporal dislocation,” by calling foods and objects ‘exotic’. This othering device serves to reduce the complexity of diversity, its historical roots and structural inequalities, and packages it in a way that is attractive to white urban middle classes and their simplified ideas of cosmopolitanism.

Overall, Charlie’s example and her strong reactions to most smells serve as an illustration of “how socio-spatial boundaries are policed through sensorial means” (Jaffe et al., 2019: 1017). The generation of these good/bad Manichean smell binaries in a heavily racialized street serve to uphold an urban sensorium in which whiteness is related to odourlessness. Methodologically, this speaks to the smellwalk’s capacity to interrogate the more affective and ephemeral locations where racialization processes operate.

#### Sounds of belonging

Niki is a middle-aged, white, educated woman, who works as a designer and has been living in Neukölln for eight years. She regularly passes through Sonnenallee on her bike on her way to other places, but she does not frequent the street itself. She characterizes Sonnenallee as having a charged, unsettling and almost aggressive atmosphere. Other associations evolve around the often-mentioned themes “chaotic traffic,” “tasty and cheap food” and “cultural diversity.” Participating in the soundwalk somewhat changed her perception as she experienced the street as more positive and interesting. Crowded restaurants for example that would have been annoying for her in another instance, felt different this time: “I perceived it more as an enrichment this time. Not as something so intrusive, unpleasant.”

Similar to positive smells that still reproduce otherness, Niki highlighted hearing women talk “African” languages and later Arabic, which she associated with being “foreign, but pleasant” and from “other worlds,” as “a bit like on vacation.” The female voices speaking a foreign language have a clear positive connotation, as an expression of living in a diverse neighborhood, which she values. However, this association largely depends on gender: when Niki hears male voices conversing in Arabic, she judges them as creating “a bit of discomfort,” they feel threatening. Thus, hearing foreign language spoken by male users of the street is a “sensory nuisance,” that ultimately differentiates between bodies that are perceived as safe versus unsafe (Fiore, 2023a: 23). Niki herself points out this discrepancy in the accompanying interview, a discrepancy that only confirms the portrayal of (young) Arab and/or Muslim migrants in public and political discourse as a nuisance and security threat (cf. Fiore, 2023a). Hearing male voices speaking Arabic leads Niki to sense insecurities (Osbourne, 2021), a sensing that is clearly influenced by more general racial orders as they exist in Germany.

The same sound - Arabic speaking voices - is interpreted differently by Yasmin, a young, highly educated woman who was born and raised in Neukölln and now works at a local organization. Yasmin’s parents migrated to Berlin from Turkey. She grew up on Sonnenallee and has lived on the Southern part of the street until recently. Yasmin makes a clear distinction between the lower, calm part of the street (south of the train station) and the busy and loud upper part that she characterizes as “two different worlds.” During the interview Yasmin talks about the changes Sonnenallee underwent and the importance of the street as an “anchor point” for people migrating to Germany, especially from the Arab world. To her, the focus on sound highlighted the diversity of the street and made it possible to gain new insights.

Confronted with the sound of people speaking Arabic, Yasmin remarks that it “raises a familiar and pleasing feeling.” She explains that the positive feeling created by hearing Arabic is not about familiarity with the language (since her parental background is Turkish), but about the fact that people feel comfortable speaking their home languages without inhibition.

Similarly, while Niki perceives the sound of cars and the bus as something that “pressures me,” Yasmin’s association with the bus sound centers around the word “home,” as she describes having lived along this certain bus line. The association with ‘home’ confirms the contributions of scholars focusing on homemaking practices of migrants (and their descendants), who have shown the relevance of sensory experiences to re-create a sense of home (Boccagni, 2023; Fathi, 2021).

Just as smell reproduces the ‘other’ through marking them as foul smelling, sound can reproduce racial hierarchies by categorizing certain sounds as unpleasant noise. Similar to Charlie’s interpretation of (positive) smells, Niki’s description of the female voices as being “foreign,” coming from “other worlds” and making her think of “vacation” is an exoticization of certain sounds. While the sounds are positively evaluated as expressions of local diversity, these sounds, in this case languages, are also placed elsewhere. Moreover, Niki’s process of meaning making reveals a gendered dimension to racialization processes that constructs the ‘male other’ as threat in contrast to an Orientalized and exoticized ‘female other’ (Yeğenoğlu, 1998). Her interpretation ties to the discursive construction of Muslim migrant men in Germany as overly aggressive, sexually predatory and dangerous to society (cf. Wigger, 2019). This gender dimension is not limited to sound: Examples of participants taking part in smell walks and linking shisha smoke to masculinity show that there is also a gendered dimension to smelling. In summary Niki’s emotionally and biographically distant account reproduces racialized and gendered perceptions of sound. She distances herself from the street while simultaneously constructing the street as distanced, manifesting a white position as hegemonic and, on an aural level, as inaudible (Stoever, 2016).

In contrast, Yasmin connects the languages she hears to a sense of belonging and feeling at home. Yasmin notes how both “people speaking German” as well as “people speaking Arabic” creates a “familiar feeling,” which is strongly connected to her personal history of growing up in the area. Her mention of the German language is an exception, highlighting that for most other participants, hearing German is an implicit, unnoticeable norm, whereby the unremarkable nature of German acts as an invisible marker of whiteness. Germany’s founding as a political entity was underpinned by the idea of *Kulturnation* whose people shared a common language and culture, working to ethnicise German citizenship and tie language to national belonging (Jarausch, 1997; Müller, 2011). While the sound of foreign languages can arouse feelings of fear, as we have seen with Niki (Lisiak et al., 2021), the sound of different languages can also create spaces where people feel at home. Yasmin describes feelings of happiness about people speaking their native languages without inhibition, or living out their religious freedom, regardless of whether it is her own native language or religion or not. In this sense Yasmin’s feeling of belonging can be seen as crossing cultural lines and might hint at the transgressive possibilities of sensory perception as stressed by Aceska and Doughty (2024), or Lisiak et al. (2021). Going further, Yasmin’s valorization of the soundscape created through different languages and religious sounds can also be interpreted as an act of resistance against a white sensorium that acts as the norm and that encroaches into more public places through processes of gentrification. Sekimoto and Brown (2020) argue that racialized subjects are much more aware of or feel social boundaries more than privileged groups. The conscious use and valorization of sounds that are connected to marginalized communities can thus be a way of reclaiming or protecting urban spaces from in-coming gentrifiers or other privileged groups and their “aesthetic sensibilities” (Summers, 2021) who will want to enforce their idea of an “ideological, aestheticised form of space” (Jaffe et al., 2019: 1017; see also Summers, 2021).

The focus on sound and the differing interpretations of similar sounds highlight how interpreting sensory input depends on biographical background and cultural embeddedness (Borer, 2013; Le Breton, 2017). The contrasting associations and interpretations of Niki and Yasmin reflect their different familiarity with and use of the street, but also their social and racial positionalities (cf. Degen and Rose, 2012). It is through daily sensing that places become familiar; Niki usually avoids Sonnenallee, even though she lives around the corner. Despite her close proximity to the street, she does not sense it daily and it remains unfamiliar. Meanwhile Yasmin’s experience is impacted by memory, highlighting Degen’s and Rose’s point that “‘everyday’, more mundane memories inflect the experiencing of built environments” (Degen and Rose, 2012: 3279).

Both highly educated women, Niki is an illustration of a typical white middle class urbanite appreciating forms of diversity that do not rupture or challenge her white sensorium. In line with wider discourses, such sensory ruptures are connected to a “sensory criminalization” (Fiore, 2023a) which challenges the presence of (young) migrantized men in urban space. Yasmin, in contrast, finds comfort in the local sensorium that *white* middle classes interpret as nuisance. Given these two participants’ similar class status, the interpretations of similar sounds as a “sensation of belonging” (Jaffe et al., 2019: 1017) versus feeling out of place, reflect their different racial positioning.

### The potential and limits of the methodology

In this final section we want to reflect on how sensory methodologies can help us to notice processes of (re-)producing whiteness, as well as on the limits of the sensory methods as applied in our project.

#### Reflexive potential or reproduction of stereotypes? From embodied experience to discursive interpretation

Drawing on two examples, we reflect on the relationship between participants’ embodied experiences and their interpretations, and how these are expressed in the group discussion. Through the research we noticed different relationships between participants’ experiences, how they chose to interpret or commit these experiences to paper, and how they discussed their observations in the group meeting. While one smell walk participant, Mira, a white woman in her 50s, wrote down several positive smells around the themes of ‘food’, ‘spices’ and ‘plants’, these positive associations did not emerge within the group discussion. Instead, Mira focused on negative accounts of Sonnenallee, describing how it was dominated by waste. Mira marked out a clear difference between her street in a neighboring, more gentrified area near Sonnenallee. Comparing the two, she calls Sonnenallee ‘embarrassing’ and finds it ‘very sad’, as it confirmed the idea that Neukölln was a trashy ‘shithole’. Mira overlooks her own sensory experiences that led to more positive descriptive interpretations of the street and reverts to dominant stereotypes in the group discussion. There could be two reasons for this outcome: first, it is an example of how “on-going ordinary, everyday memories” (Degen and Rose, 2012: 3279) mediate people’s experience and interpretation of the sensory walk. Her (negative) memories and emotions, which are clearly related to the topic of ‘waste’ overshadow any positive sensory experience that Mira clearly has had. Second, her focus on negative associations is also possibly an expression of ‘cultivated learning’, underlining the influence of media discourses and cultural reference points. While sensory experiences may disrupt Mira’s usual interpretations of Sonnenallee, negative images come to dominate the discussion, signaling how difficult it is to move beyond learned and embodied, already incorporated, ways of sensing.

This recollection of stereotypes from participants like Mira connects to how everyday boundary drawing practices work. Blokland et al. (2023) have described how familiarity is key to social interactions and building trust in urban spaces, yet when people come to associate a street like Sonnenallee with bad smells or odors, or otherwise exoticize it, they can avoid using this street in their daily lives. This creates a lack of familiarity which can become connected to a lack of trust, so that stereotypes eventually become translated into avoidance and less contact with the often stigmatized and marginalized population that lives on Sonnenallee. This further cements media portrayals of the street as a problematic threat.

Another research participant shows us how sensory experiences could instigate reflexivity. Liam, a young student who was born in Armenia but moved to Germany with his parents at an early age, was among the few participants who also reflected on how potential prejudices might influence his perception of the street. While the associations he noted before the walk were rather stereotypical, and the smells he noted also included associations with “Orient” or “vacation,” in the discussion Liam critically assessed his interpretations: “Well, of course I had to realize how big the influences of stereotypes are, whether you like it or not. And that these certain boxes “(Schubladen)” are also opened when smelling.” Liam goes on to describe the smell of a “mixture of perfume, tobacco, very flowery, ultra-fresh and cold smell” that reminds him of vacation in Turkey or Arab countries. This association originates through his personal experience, which is why he judges it to be “not a prejudice or anything.” Liam did smell these things on holiday, and there is clearly crossover between Turkey, the Arab world and Sonnenallee, a thoroughfare which shows how the world can be located on a street (Hall, 2012). Yet at the same time Liam acknowledges that the smells he observes still fit with learned prejudices, as they invoke certain associations of multiculturalism (“Multikulti”). Thus, while his reflexive comment was unusual, a sensory focus certainly has the potential to incite contemplations about one’s own experience and what associations are employed. While we observed, as expected, ample processes of boundary drawing, it seems that sensory experiences also hold potential for boundary crossing. The examples seem to suggest that a major mechanism for boundary crossing consists of moving beyond learned, cultivated ways of interpreting sensory input.

We are left with areas that require further attention and exploration. We need to develop a better understanding of how acts of boundary crossing could also be understood as ‘positive’ racism. Where do we draw the line between an openness to everyday multiculture and more seemingly positive acts of racial othering? We feel that while our method of a single walk through the street is effective for generating a range of affective observations, it is less useful in terms of capturing how boundary crossings take place over space and time. For this, ethnographic methods would be a more effective tool. Through the project, we have also tried to discern how micro-level embodied sensory experiences tie to the more macro or structural level. How can we and should we understand these inter-connections? Our project shows how the act of sensing – something that can often be portrayed as value-free or primordial – is something always already informed by and intricately connected to the social world. Sensing is shaped by positionality, dominant discourses, and previous experiences. Yet the research also hints to the potentiality of the sensory to disrupt discourses and open windows to new perspectives.

#### Research on a highly mediatized street

The street we focused on is a highly diverse street that, as described earlier, is heavily mediatized. The district of Neukölln and Sonnenallee are widely known across Germany, which gives the area a symbolic position in the national imagination. Anxieties around issues of migration and integration are squarely projected onto the street, which is evidenced via newspaper headlines that frequently describe the area as a *Brennpunkt* or problem area, while books with titles like *Brennpunkt Deutschland: Armut, Gewalt, Verwahrlosung - Neukölln ist erst der Anfang* (Lieke, 2022) (Problem area Germany: Poverty, Violence, Neglect - Neukölln is only the beginning) cement an image of the area as a malignant blight on the national landscape. The diversity of Sonnenallee could be described as contested diversity, in contrast to “commonplace diversity” (Wessendorf, 2014). Yet other more sympathetic media portrayals are also in circulation which highlight the street’s diversity as something positive and innovative, referring to the different music, food, and cultural activities available in the area. Interestingly, while Sonnenallee is clearly over-mediatized, it is not a place that could be called over-researched, as scholarly texts on urban transformation and/or migration in Berlin focus on other districts such as Kreuzberg or Prenzlauer Berg.

The negative, racialized image of Sonnenallee invites reflections on how we can do research without doing harm. This applies to the research context itself: several of our (white) participants, walking around with a clipboard, noting their impressions, raised suspicion among local shopkeepers who asked whether they were part of the regulatory office - a branch of the local state that is regularly present on Sonnenallee and plays a role in its criminalization. Additionally, doing research without harm also concerns how the data are analyzed and presented. Given the negative picture of the street, plus our own background as white, privileged researchers, it is important not to further compound the exoticization of the street and its residents/business users.

This necessitated reflection about how we as researchers can do necessary research, without further exacerbating feelings of surveillance and over-policing on the street. The marking out of areas adjacent to Sonnenallee as *Kriminalitätsbelastete Orte* or crime hotspots, which gives the police wider powers to stop and search anyone within these areas, undoubtedly contributes to feelings of surveillance and suspicion. We did not wish to duplicate this gaze of surveillance and, particularly in the wake of the war in Gaza, tensions on the street were running so high that we decided to curtail our interviews with shopkeepers. The atmosphere was uncomfortable and we did not want to contribute to a feeling of discomfort, which was already created through media and political discourse, coupled with the visibly increased policing of the street.

Part of our attempt to decrease harm in the research process was through recruiting a diverse sample. We initially recruited via neighborhood-based platforms and student lists, yet quite predictably, this led to a sample of white, privileged participants from both within and outside Neukölln. Our second step included contacting people from local organizations, which helped increase the sample’s diversity, particularly in terms of respondents’ ethnic and class backgrounds. Our third approach to increase diversity by interviewing local shopkeepers was only partially successful. While these brief interviews helped us gain a better understanding of their concerns and perceptions of the street, including struggles against its vilification, they did not allow an understanding of how shopkeepers sensed the streets.

Apart from language issues, puzzled reactions to the questions about smell and sound might have two reasons: first, they might highlight how uncommon it is to speak about these senses, and the difficulty of spontaneously verbally articulating sensory phenomena. This would support the necessity of the smell or sound walk as a method to access this dimension, whereby time and solitude enabled participants to notice these phenomena. Yet having the time to participate and the habitus to feel comfortable writing responses and discussing them in German acts as a mode of exclusion from the research.

However, the reactions might also highlight a Western bias about how certain sensescapes are perceived as remarkable, given their divergence from the norms of odourlessness and silence, which are connected to whiteness. For the immigrant entrepreneurs on Sonnenallee, these ‘diverging’ sensory regimes are not remarkable, as they are immersed in them on a daily basis. it could be difficult to talk about these regimes, just as white Germans might find it difficult to talk about the norm of ‘quietness’ in a typical white middle-class area. Thus, what is *sensational* for some could be sensorily unremarkable for others (Seremetakis, 1994).

Our own involvement in the street and our limited language capacities are themselves a practice and reproduction of whiteness. We might have had different experiences had one of our team members shared the languages spoken by the local shopkeepers and possibly shared their regional background. Thus, the context of the research prompted reflections on our own impact on the street and its users while doing research, about how we produce knowledge, which categories we use and how we could reproduce or avoid the reproduction of these categories through our research practice.

### Summary and discussion

This paper has attempted to illustrate the purchase of sensory methods to interrogate how the urban is perceived, classified and how social boundaries are being made or broken. Boundary making via the senses was evident through the accounts of our research participants, whereby smell and sound worked to reproduce ‘the other’ through both positive and negative forms of racialization and classification. Regardless of the smells or sounds being deemed positive or negative, they were still placed firmly outside of the locality and viewed as being located elsewhere - despite their long-term presence on Sonnenallee. How smells and sounds were perceived and described tied heavily to the participants’ social and racial positionings, their use of and familiarity with the street, and the more general media and political discourse about the place. Overall, the paper clearly highlighted the purchase of introducing the senses into the study of urban diversity. While much of the scholarship on urban diversity looks at interactions or fleeting encounters, our research has foregrounded how diversity is sensed, and how sensory experiences and their interpretations more often than not reproduce and confirm heavily racialized stereotypes. Following insights from sensory urbanism scholarship, our study clearly points to the ‘visceral micro-politics’ of boundary drawing and exclusion through sensory experiences. Smells and sounds that are interpreted as a nuisance, or as too strong, too loud, or too foreign, clearly influence which kind of diversity is to be celebrated and which kind of diversity is to be feared. While many of the neighborhoods surrounding Sonnenallee are gentrified, Sonnenallee itself still seems to resist this pressure - with a predominance of ‘ethnic businesses’ that do not specifically promote a ‘gentrifier-friendly’ atmosphere (except the occasionally advertised vegan Döner). Thus, Sonnenallee is not yet a “space of aestheticized multicultural urbanity” (Fiore and Caradonna, 2025: 95) that is attractive to the urban white middle classes. Therefore, for white middle-class users of the street, Sonnenallee seems predominantly sensational, and many of the smells and sounds challenge the ‘white sensorium’ that is characterized by sounds and smells constructed as neutral. For the white participants of the study, whether local or not, consciously walking and sensing along Sonnenallee was an immersive, singular experience that ultimately (not only, but predominantly) highlighted previously learned categories of who and what belongs locally versus other spatial locations, as mediated through the senses. While being exposed to a different context, outside daily routines, can lead to feelings of solidarity (cf. Jaffe et al., 2019), in our cases, sensory ruptures dominated. This overwhelmingly led to the confirmation of stereotypes and the feeling of social distance between the street and its inhabitants (cf. Jaffe et al., 2019).

An open question remains regarding how reflexivity, or a sensory learning, can be initiated through sensory methodologies. For such a process to happen, methodologies such as sensory walks and subsequent (group) discussions would need to be expanded to also confront participants with their own prejudice, initiating ways of sensory learning, of ‘knowing differently’ (cf. Harris, 2020). It is an open question how this is possible, and how, in that process, an ethics of discomfort can be a part of an ethics of care (Zembylas, 2017) that is necessary to break away from long learned and incorporated ways of sensing and sense-making. While race and racism are often approached in their expression as embedded in societal structures, our research has highlighted how race/whiteness is embodied, how it turns into a social reality through what is sensed through the body, hence clearly highlighting how the focus on the sensory - and sensory education as an outcome - needs to be part of the study of how race is produced, particularly in the context of increasing urban diversity.

## Data Availability

The datasets presented in this article are not readily available because the data we gathered is sensitive, which is why it is not an any public repository. Requests to access the datasets should be directed to barwicch@hu-berlin.de.

## Footnotes

^1^ https://mediendienst-integration.de/fileadmin/Dateien/Expertise_Sonnenallee_Stock_Mediendienst.pdf
